# Accelerating problem-solving capacities of sub-national public health professionals: an evaluation of a digital immunization training intervention

**DOI:** 10.1186/s12913-022-08138-4

**Published:** 2022-06-02

**Authors:** Karen E. Watkins, Lorilee R. Sandmann, Cody Aaron Dailey, Beixi Li, Sung-Eun Yang, Robert S. Galen, Reda Sadki

**Affiliations:** 1 Learning, Leadership and Organization Development Program, Mary Frances Early College of Education, University of Georgia, 850 College Station Rd, Athens, GA USA; 2 College of Public Health, University of Georgia, 100 Foster Rd, Athens, GA USA; 3 Office of Health Affairs, West Virginia University, 64 Medical Center Dr, Morgantown, WV USA; 4 The Geneva Learning Foundation, Av. Louis-Casaï 18, Geneva, Switzerland

**Keywords:** Immunization, Training, Problem-solving capabilities, Capacity-building, Peer learning, Sub-national public health professionals, Evaluation

## Abstract

**Background:**

This article reports an evaluation of the *Immunization Training Challenge Hackathons* (ITCH)**,** invented by The Geneva Learning Foundation (TGLF) for national and sub-national immunization staff who strive to develop the knowledge and capacity of others to improve immunization program performance. ITCH, a fully-digital program focused on networked collaborative problem-solving between peers, provided an “opt-in” activity for learners in the Teach to Reach (T2R) Accelerator Program designed to improve training effectiveness in the immunization sphere.

**Methods:**

Conducted by a team from the University of Georgia, this mixed method evaluation consisted of thematic analysis of recorded sessions and open-ended comments; and statistical analyses of application and follow-up survey data. The evaluation focused on what was learned and how ITCH participants implemented what they learned**.** Key stakeholder interviews provided supplemental data about program intent and results. ITCH consisted of 17 30-min sessions held in 2020, in English and French, with 581 participating at least once out of 1,454 enrolled in the overall program. Challenge owners and respondents came from 15 African and Asian countries and spanned different roles with differing scope.

**Results:**

Over 85% [*n* = 154] of survey respondents [*n* = 181, a 31% response rate] indicated they were able to implement what they learned from the ITCH sessions. A majority [*n* = 139, 76.7%] reported finding the sessions useful. Issues with poor connectivity and the timing of the live meetings impeded some in their ability to participate, a problem compounded by consequences of the pandemic. The ITCH process constituted of learning or coming to consciousness simultaneously of four types of learning — participants realizing how much they could learn from each other (peer learning), experiencing the power of defying distance to solve problems together (remote learning), and feeling a growing sense of belonging to a community (social learning), emergent across country borders and health system levels (networked learning).

**Conclusions:**

Based on evaluation findings, it was concluded that ITCH demonstrated an effective scalable, informal, non-didactic, experience-led, fast-paced, peer learning design. A focus on community engagement and developing brokering skills was recommended.

**Supplementary Information:**

The online version contains supplementary material available at 10.1186/s12913-022-08138-4.

## Background

The growing complexity of international immunization programs demands a health workforce with strengthened skills and competencies. Yet, some healthcare workers in low- and middle-income countries lack the necessary skills to perform essential tasks in response to the needs of the local population [1]. The typical training approach, usually a short, offsite, in-service cascaded training, can no longer effectively address the challenges health workers might face in implementing immunization interventions [2]. The cascade approach to training where training begins at the top of a hierarchical chain, with trained individuals then training their direct reports, is known to become weaker and weaker the farther down the chain one goes and the farther away one moves from the subject matter experts who initially conducted the training [3]. This approach is cost effective for reaching a large group of people, but less effective for actually yielding performance at the local level [4]. Indeed, looking across 15 educational interventions in training healthcare workers, only group processes including peer review had moderate effects and community case management had moderate to large effects [5]. Training and workshops had mixed results. This study evaluated a promising scalable, digital approach developed by the Geneva Learning Foundation as an alternative to conventional forms of training increasingly recognized as ineffective.

The Geneva Learning Foundation (TGLF) has partnered with the Bill & Melinda Gates Foundation (BMGF) and the World Health Organization (WHO) in testing and scaling more efficient and less disruptive ways of embedding learning and capacity development into work itself rather than through traditional on-site training. Teach to Reach (T2R), a Gates Foundation initiative focused on improving training effectiveness, funded TGLF to transform T2R from a traditional conference format to a digital learning platform.

This study is an evaluation of the learning outcomes of TGLF’s approach using the Immunization Training Challenge Hackathons (ITCH) as a microcosm or “learning fractal” of the larger TGLF approach to capability and leadership development through peer learning. The term “hackathon” was used to convey the idea that this was fast-paced problem solving. The goal of these optional sessions was to help participants break through barriers in their own contexts by learning from each other about immunization training challenges and solutions. The formal learning objectives of the Teach to Reach Level 1 certification were to develop an action plan to improve an immunization training program in relation to an immunization challenge, and then to peer review the plans of other participants to help others improve. Within that larger process, the ITCH sessions were designed as open spaces with both content and context defined by participants, workshopped just-in-time by peers sharing practical experiences and solutions. The evaluation investigated these sessions to explore what facilitated learning and to what extent this informal learning approach lead to knowledge creation and potential new actions that contribute to or correlate with improved immunization program performance (outcomes).

## Methods

This was a mixed methods study which drew on the following data: T2R course artifacts, including 3,733 T2R applications, challenges submitted by 303 T2R participants, and 17 ITCH session recordings. Inductive thematic analysis was performed to identify the critical issues in immunization and training [6]. Transcripts were reviewed by two coders independently to increase trustworthiness and the evaluation team was made up of both public health and learning scientists. While TGLF leadership provided suggestions and contextual information to inform the research methodologies, the evaluation team maintained technical control over the evaluation research process. Statistical analyses using R statistical package of selected components of the applications, submitted challenges, and ITCH sessions augmented the inductive qualitative analyses and ensured data triangulation. A follow-up survey was developed by the evaluators to examine the impact of ITCH sessions to augment the above data provided by TGLF. The human subjects approved survey (in English and French) was distributed through Qualtrics to 581 participants of ITCH sessions. Challenge owners, respondents, and attendees were invited to take the survey and assured their responses would be anonymous. One hundred eighty-one participants (31 percent) completed and returned the survey. The following questions were addressed in this study:

1. What immunization outcomes and effects did ITCH initiative participants identify?
2. What informal and incidental learning was fostered through the ITCH sessions?

As a supplement to the data sources described, semi-structured stakeholder interviewers were conducted to ascertain the goals and intentions of the approach by the ITCH designers and facilitators and their assessment of the impact of the ITCH session. Thematic analysis was completed on these data as well.

This evaluation adopted an adapted complexity approach [7]. According to Newton-Lewis [8], digital interventions need to be sensitive to the local context and systemic predicaments of health workers to understand the complex systems in which they work. In this study, ideas from complexity theory were used as sensitizing concepts to analyze the data, looking particularly at adaptation and emergence to identify what emerged from this open-ended approach within the complex relationships within and between ITCH sessions, the wider program, and the work environments of participants. Finally, the evaluation approach was collaborative, working with TGLF to identify the goals of the study and to design the survey to assess results [9]. O’Sullivan (2012) notes that collaborative evaluation believes that ongoing collaboration between evaluators and program staff leads to “stronger evaluation designs, enhanced data collection and analysis, and results that stakeholders understand and use” [9].

### Program description

As part of the T2R program application, 3,733 participants were encouraged to identify an immunization training challenge they were facing in their roles. Of these, 303 did. Specifically, they were asked their name, role, level of employment, and country and to describe the training challenge, problem, or dilemma they were trying to solve. Drawing from the problem analysis process used in action learning (O’Neil, Watkins, Marsick, 2010) [10] participants answered these questions about the problem or challenge:

- Identifying and describing the challenge
  - How did you identify this challenge?
  - What have you done to learn more about it?
  - What strategies or solutions have you tried or would you consider?
  - Who or what has helped you?
- Reflecting on the challenge
  - Was there anything that surprised you?
  - Have you had any Eureka moments?
  - Is there anything else that we need to know?

**Fig. 1:**
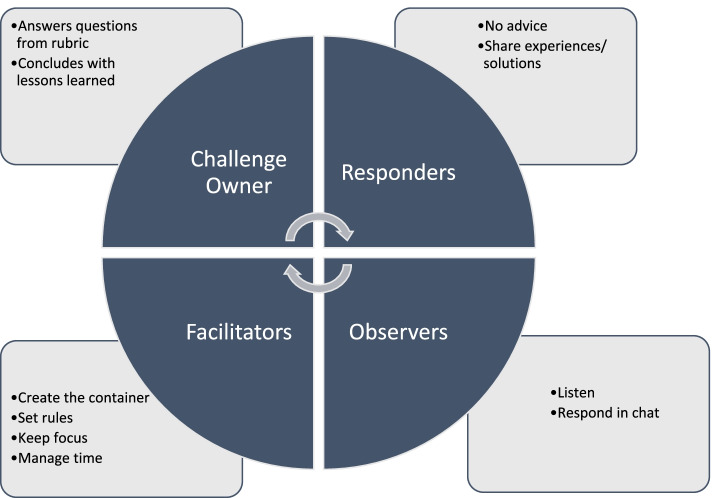
ITCH 30-min microlearning approach

During each ITCH session, a “challenge owner” was identified and the information submitted in their application shown on screen to all participants. Peers were then invited to share their experiences in relation to this immunization training challenge. Figure 1 depicts the key elements of the Immunization Training Challenge Hackathon.

Transcripts from the ITCH sessions, information from initial applications and from a certification database were linked into a single database. From March 17 to April 17, 2020 for Anglophones and from April 6 to May 11, 2020 for Francophones, ITCH sessions were held twice weekly as interactive, virtual video conferences. A total of 17 sessions were held; nine in English, eight in French. A typical 30-min session involved one T2R participant presenting their challenge and the other attendees problem solving or providing counsel from their own experience or context. Responders at each session numbered from 2–5. Peers were encouraged not to be prescriptive (“you should do this”) in their responses, but, in a fast-paced “hackathon” manner, to share their own experiences (“This happened to me”), efforts (“I tried this…”), and initiatives (“Here is how it turned out for me…”) relative to the challenge presented. Facilitators kept the momentum and helped focus the learning. In addition to the challenge presenters and respondents, 526 scholars were online as active listeners. In total, 581 participated in the ITCH sessions. Table 1 illustrates the challenges presented and responses.

**Table 1:** Selected training challenges and responses

Owner Title	Challenge	Responses	
Subnational MoH staff member in Côte d’Ivoire	Cross-border immunization campaign	1	Cross-border meetings to identify unvaccinated children and solve other issues
		2	Cross-border meetings to organize and synchronize efforts. WHO and UNICEF’s support in the collaboration
		3	Track children with different colored cards depending on the region of vaccination
		4	Involve community representatives in cross-border meetings
		5	Cross-border collaboration to ensure vaccination of all children. Identify permanent parts of populations and considering issues like cultural differences, language barriers and other situation-dependent problems
WHO HQ staff in Democratic Republic of Congo	Data quality	1	Additional survey to help re-estimate of data targets
		2	Vaccination campaigns conducted alongside efforts to count children and target populations
		3	Use teams to divide the responsibility of administering the vaccine and filling out records at the same time
NGO staff member in Kenya	Effective training in a resource constrained situation	1	Interactive e-learning module with a certificate of completion at the end
		2	Different stakeholders joining hands to address the challenge together
		3	Provide on the job training opportunities like mentorship and training integration
MoH staff member in Democratic Republic of Congo	Communica-tion issues and vaccine hesitancy from community	1	Involve community members in campaign and train them to help out
		2	Demonstrate to spiritual and religious leaders that vaccine is safe, and vaccinators are properly trained
		3	Different vaccination programming for different regions

It is important to note that COVID-19 struck in the course of these sessions—with significant disruption to training and immunization schedules of participants. The support offered challenge owners during this time, the responsiveness to concerns, and the sense of community fostered by the TGLF approach were particularly suited to the needs of these individuals at this time which is evident in our findings.

## Results

We begin by describing the demographics of the participants in the ITCH sessions. Results are then presented by evaluation research question. Since our data analysis spanned all T2R scholars’ applications and completions as well as all ITCH participants’ data from the 17 sessions as well as a sub-set of participants who responded to our follow-up survey, it was important to ascertain that the two sub-sets were similar in composition to the overall groups. Overall, T2R participants mainly worked or focused on immunization with West and Central African countries, although a total of 104 countries spanning the globe were represented. Similarly, ITCH challenge owners and respondents came from 15 African countries and spanned a range of roles, from WHO headquarters to country office staff, from national Ministry of Health (MoH) staff to MoH health facility staff, as well as consultants and NGOs. The majority of respondents worked at the regional or district level while the challenge owners clustered at the district or other sub-national levels. Challenge owners had less experience in training than responders. Results from the survey follow [See Supplementary Table 1].

The survey consisted of four parts: demographic data, immunization concerns, questions about the ITCH sessions, and questions about potential issues and recommendations. The mean number of sessions attended was 4.39. The issues participants brought as a challenge most often were dealing with the local community (resistance, misconceptions, cultural barriers, access) followed closely by issues related to the logistics of organizing and managing training.

Overall, participants found the sessions very useful [median of 6.0 on a 6-point scale (IQR = 5–6),]. In fact, over half (*n* = 106, 58.6%) of respondents found the sessions extremely useful. Participants said they were able to implement the ideas shared during the ITCH sessions (mean = 5.0 (IQR 4–6); median 5.0). While the survey was self-reported perceptions, these results indicate at least a belief by participants that what was learned in these sessions was useful and implementable. Finally, 83% of ITCH participants [*n* = 421] received a certificate of completion of the T2R program compared to 60% of all other T2R participants [569 of 946 scholars]. We also asked if they had any difficulties that might have affected their ability to participate in the ITCH sessions. Issues of poor connectivity were mentioned by 34.3% (*n* = 62) of respondents and almost 36% (*n* = 65) had issues with the timing of the ITCH sessions.

Our final research question asked what was learned through these virtual sessions. Survey responses to our open-ended question: “What did you learn from participating in the ITCH session?” ranged from comments on specific strategies implemented to learning from the design of the hackathon itself. Learning from peers was the most frequently mentioned strategy. Participants mentioned the value of experiences shared by others, social networking, enhancing communication skills while in learning sessions, and the peer learning experience per se. Respondents valued the opportunity to see and experience learning and training modeled using new digital approaches. The use of new technologies for remote learning was an important learning outcome. Respondents said they discovered useful solutions to immunization training challenges through ITCH.

*Learning from peers* was the most frequently mentioned strategy. Participants commented:

- *The experience sharing, the discussion on a peer challenge, the different interventions (facilitators, peers) allowed me throughout the session to improve my work (the way to performance). This sharing was very rewarding, because we learn a lot by helping others and / or by sharing with others.*
- *I learned to work in a team, that is to say not to die in silence, to consult others in anything to have the solution.*

*Respondents valued the opportunity to see learning and training modeled using new digital approaches.* Learners said they were exposed to new technologies during the sessions, and learned how to apply those technologies in their practice.

- *That I could organize training without looking for external funding. How to use the means available to solve a vaccination or general health problem without locking the actors in a space.*
- *I appreciated the power of technology in organizing training on scale.*

Many of the respondents said they learned useful solutions through this course.

- *I have learned the difficulties are almost identical in all member countries of WHO Africa but the management of these problems and functions of each staff [differ].*
- *I learned to find a problem and implement it*

What is evident across these responses is that participants felt the sessions were very valuable, —learning from each other [peer learning], seeing a virtual problem-solving session in action [remote digital learning], and the sense of community [social learning] that emerged from feeling the problems each brought to the sessions were shared across country borders [network learning].

From a complexity science perspective, we were interested in adaptations and emergence. How did developers of the ITCH process adapt to respond to the unique conditions of their context – low and middle income, largely African countries, large numbers of sub-national staff amidst a global pandemic. Table 2 offers our findings regarding the outcomes as we heard them expressed by the developers of the ITCH program and what we found both among those intended and those that emerged out of the complexity of the situation.

**Table 2:** Evaluation of ITCH outcomes—both intended and emergent

Planned Outcomes	Intended Outcomes Found	Emergent Outcomes
Reach to sub-national level	Increased proportion of sub-national participants	Regional and national participants were more often responders
Generate relevant learning	Learners identified personal challenges; *n* = 139, 76.7% found the sessions useful	Since many had the same issues, observers also found useful ideas
Blend formal, informal, open-ended digital learning approaches	Modeled more effective approaches to digital training	Many [*n* = 154, 85%] said they could replicate ITCH in their setting
Introduce and increase comfort with new technology	Participants used Zoom, learning. foundation, Campuswire, etc	Frustration where connectivity was not affordable or limited
Privilege peer coaching (giving and receiving feedback)	Peer support was most valued aspect	High affective response; high satisfaction
Increase training impact	*n* = 154, 85% were able to implement what was learned	High level of transfer of skills

## Discussion

Looking across the data from this evaluation, we noted that ITCH demonstrated an effective scalable, informal, experience-led, fast-paced, peer learning design. We conclude this for a number of reasons. First was global reach—this optional component involved almost 600 people from 15 countries. Furthermore, it brought together people from various roles and system levels. As a WHO staff member commented, *“I didn't have enough knowledge to be able to [know] how to handle the immunization training challenge that I was facing as a practitioner, but based on the experience shared by somebody in India or Kenya, I can learn how to handle the situation in my country.”* The program, as a stakeholder noted, was another vehicle for sharing successes among peers: *“The Bhutan person mostly thought he had done something good that he wanted to share.... He thought it was something that could be useful.*” Perhaps most importantly, the program modeled intensive brief virtual coaching that could readily be implemented in participants’ workplaces. There is a strong sense that as Marshall McLuhan (1964) said [11], the medium is the message—or at least a significant part of it.

Learning from and with each other, in community, was paramount. People wanted to build their professional network. They asked for contact information of participants, rated peer learning groups as their most desired follow-up to these sessions, and wrote comments about the importance of not “*dying in silence*.” We noted that the impact of these sessions was at least as much affective as it was cognitive. As one stakeholder mentioned, “*…maybe just having a community group that, you know, is your tribe, like, these people are living my life, too, and I'm not alone and it's just a relief to know there are other people who are in this with me.”* One person commented, “*ITCH sessions are really support sessions for me*.”

### Recommendations

Two primary logistical concerns—timing and connectivity were emphasized in the survey. While connectivity will not be readily solved without significant capital expenditures, the timing of the sessions could be addressed by offering the sessions at varied times on different days of the week. A more significant recommendation responds to the many examples given in the case challenges studied here of the need to work closely with the community in order to overcome vaccine hesitancy, to implement microplans, etc. By helping immunization professionals recognize and expand their role as boundary spanners between their public health organization and the community they hope to reach, they can enhance what is already a significant tool in their practice—community engagement.

Experiences in community involvement and community leader engagement were shared in various ways by respondents of the ITCH sessions. For example, one respondent noted “*when the microplan is done at the local level with the community leaders, it would be more easily carried out. Now, every community leader has to register documenting the newborn babies, which can be used to support the microplan*” In the last two decades, public health efforts have increasingly employed community engagement to improve overall health outcomes as successful initiatives evolved into lasting collaborations (https://www.cdc.gov/globalhivtb/who-we-are/resources/keyareafactsheets/Ensuring-Quality-Health-Systems-and-Human-Resources_1.pdf).

Community engagement is a way of doing agenda setting, design and delivery, implementation, interventions, and change. It means work is not done “in” or “for” communities, but rather “with” community leaders, both formal and informal. Working from a community engagement conceptual framework, developing, implementing, and evaluating training programs would be done with community partners involvement (https://www.atsdr.cdc.gov/communityengagement/pdf/PCE_Report_508_FINAL.pdf). It is recommended this start with a strong community voice or presence joining in the planning and discussions of the training. It would also include developing the requisite skills and abilities in immunization practitioners to identify and work with the community partners, local or national, to achieve mutuality and reciprocity.

We recommend highlighting the functions and roles of immunization practitioners, from frontline sub-nationals to administrative nationals, as critical boundary spanners, connecting with each other based on need and purpose. As noted by one ITCH challenge owner dealing with resource constraints, a subnational minister of health staff member in an African country indicated … “*in terms of resource mobilization, it is important to get different, key stakeholders to join hands and address the challenge*.” Boundary spanners are bridgers between an organization and their community partners. Friedman and Podolny (1992) [14] note two major functions of boundary spanners:

1. To convey influence between constituents and partners: negotiating power and balance among the institution and community partners to achieve mutual objectives.
2. To represent the perceptions, expectations, and ideas of each side to the other: *performing teaching and learning functions to promote mutual understanding among organizations or groups (italics ours).*

Within those broad functions, there are overlapping boundary spanning roles present in the diverse ITCH participants, similar to those described by Weerts and Sandmann (2010) [15]: community-based problem solvers, technical experts, institutional internal advocates, and external champions. Explicit activities (including but not limited to ITCH sessions) to enhance respective boundary-spanning roles would highlight the concept of boundary spanning and its importance; assist public health workers in appreciating their respective boundary spanning opportunities and roles; and learn better how to function within them and specific skills involved in such roles, including how to resolve potential conflict among spanners.

### Limitations

We recognize that the study has several limitations. First, the evaluation used a self-reported instrument, which may introduce response bias. While a response rate of 31% was sufficient for these analyses, a higher rate of response may have given a more complete picture. The sample may represent self-selection bias in that both ITCH participants and survey respondents had more immunization experience than among T2R applicants. The survey study was conducted over six months following the ITCH experience and may have been somewhat early for determining program impact and somewhat late for recalling specific concerns about the experience. The results are robust in terms of internal validity and reliability given that the survey sample was comparable to the full population of Teach to Reach attendees as well as those who attended the ITCH sessions. In terms of external validity, to the extent that the T2R participants reflect the larger immunization personnel population they are but as we note, both groups are skewed toward African countries and low and middle income countries- a target population of this program.

## Conclusion

The data reflected in this evaluation demonstrate that participants found value in the ITCH sessions through four types of learning: peer, remote digital, social, and network. We have sought to illustrate what was valuable from participants’ perspectives and also to suggest the pedagogical and process elements that appear to have ensured value creation from our perspective as learning scientists and public health scholars. What remains is to explore further variations of this process to continue to evolve an intense and productive digital learning experience.

While the general issues with vaccine delivery, uptake, and training outlined here are common, they were exacerbated by the current landscape of COVID -19. Many of the solutions suggested were not new and simply reminded others what WHO has recommended for dealing with these common recurring problems. Nevertheless, it is significant that participants identified *for themselves* and *with each other* which approaches were most relevant to a specific context and how to adapt them, as this has proven the most difficult part of turning global recommendations into local action. Training challenges were organically and intrinsically tied to immunization challenges. While it is overly simplistic to imagine a training codebook such that if you have this immunization challenge, these training approaches are most effective, it was clear these individuals developed several successful approaches and the hackathon approach was effective in surfacing and sharing them, recognizing both commonalities and differences.

As fully-digital programs will remain an important delivery modality for global education and training, the process used in the ITCH hackathons has generalizability to other fields and audiences in similar peer, remote, social, and network learning. In the survey responses, participants reported adaptations and implementation of the process underway in their settings.

We are mindful this evaluation looked at a small “fractal” or microcosm of a dynamic system with many moving parts—as Pendleton-Jullian and Seely Brown (2018) [16] said, “impact requires achieving resonance between new things made, new actions taken, and the contexts in which these new things and actions reside—contexts that don’t stand still.” Given the pandemic amid this process, the contexts in which these individuals work was definitely fluid and COVID-19 created urgency about solving persistent immunization issues such as cold chain and hesitancy while also increasing willingness to try digital learning approaches. Despite the difficult context, this small slice of activity promoted a surprising level of learning in a brief time.

The learning sciences have sought but seldom found the level of evidence-based practice that distinguishes health research since the sheer complexity of potentially influencing and intervening variables has confounded causal studies. Yet studies such as this one can offer best practices and practical wisdom where there is no script for what to do. Complexity theorist Alicia Juarrero notes that to learn something new, we need to restructure the “space of possibility.” The ITCH sessions are such a space of possibility [17] [18].

## Supplementary Information

**Additional file 1:** **Supplementary Table 1.** Demographics. 

## Data Availability

Data are available from the corresponding author upon reasonable request at kwatkins@uga.edu.
