# Supplementary material for: Accelerating problem-solving capacities of sub-national public health professionals: an evaluation of a digital immunization training intervention
Source: BMC Health Serv Res. 2022 Jun 2;22:736. doi: 10.1186/s12913-022-08138-4 (PMC9161754; doi:10.1186/s12913-022-08138-4)
Supplement: Supplementary file 1 — Additional file 1: Supplementary Table 1. Demographics. [file 12913_2022_8138_MOESM1_ESM.docx]

**Supplementary Table 1** Demographics

|  | **Applications** | **T2R Scholars** | **ITCH Participant** | **Survey Respondents** |
| --- | --- | --- | --- | --- |
| n | 3618 | 1399 | 560 | 181 |
| What is your organizational affiliation? (%) |  |  |  |  |
| Global Immunization Partner Staff | 49 (1.4) | 22 (1.6) | 9 (1.6) | 7 (3.9) |
| World Health Organization HQ Staff | 18 (0.5) | 5 (0.4) | 2 (0.4) | 1 (0.6) |
| Consultant currently working for WHO/UNICEF | 281 (7.8) | 137 (9.8) | 84 (15.0) | 21 (11.6) |
| WHO Regional or Sub-Regional Office Staff | 66 (1.8) | 33 (2.4) | 8 (1.4) | 2 (1.1) |
| WHO Country Office Staff | 172 (4.8) | 82 (5.9) | 27 (4.8) | 0 |
| National Ministry of Health Staff | 996 (27.5) | 329 (23.5) | 141 (25.2) | 44 (24.3) |
| Sub-National Ministry of Health Staff | 837 (23.1) | 360 (25.7) | 128 (22.9) | 48 (26.5) |
| Non-Governmental Organization Staff | 456 (12.6) | 175 (12.5) | 66 (11.8) | 24 (13.3) |
| Student or Academic | 224 (6.2) | 62 (4.4) | 18 (3.2) | 5 (2.8) |
| Other United Nations Staff | 73 (2.0) | 37 (2.6) | 19 (3.4) | 5 (2.8) |
| Other Consultant | 135 (3.7) | 51 (3.6) | 23 (4.1) | 7 (3.9) |
| Other | 311 (8.6) | 106 (7.6) | 35 (6.2) | 8 (4.4) |
| Missing | 0 | 0 | 0 | 9 (5.0) |
| At what level of the immunization system do you mainly work? (%) |  |  |  |  |
| Global | 55 (1.5) | 20 (1.4) | 10 (1.8) | 4 (2.2) |
| Regional | 266 (7.4) | 98 (7.0) | 40 (7.1) | 22 (12.2) |
| National | 709 (19.6) | 296 (21.2) | 120 (21.4) | 41 (22.7) |
| Province, State, Zone, or Region (sub-national) | 883 (24.4) | 399 (28.5) | 169 (30.2) | 37 (20.4) |
| District (or equivalent) | 1045 (28.9) | 397 (28.4) | 157 (28.0) | 52 (28.7) |
| Health Facility (or equivalent) | 660 (18.2) | 189 (13.5) | 64 (11.4) | 17 (9.4) |
| Missing | 0 | 0 | 0 | 8 (4.4) |
| How many years of experience in the field of immunization do you have? (mean (SD)) | 7.28 (5.13) | 7.86 (5.08) | 8.45 (5.06) | 9.16 (4.98) |
| How many years of experience in the field of training do you have? (mean (SD)) | 6.92 (4.97) | 7.39 (5.01) | 7.54 (4.98) | 7.34 (4.63) |
